# Automatic method for the dermatological diagnosis of selected hand skin features in hyperspectral imaging

**DOI:** 10.1186/1475-925X-13-47

**Published:** 2014-04-22

**Authors:** Robert Koprowski, Sławomir Wilczyński, Zygmunt Wróbel, Sławomir Kasperczyk, Barbara Błońska-Fajfrowska

**Affiliations:** 1 Department of Biomedical Computer Systems, University of Silesia, Faculty of Computer Science and Materials Science, Institute of Computer Science, ul. Będzińska 39, Sosnowiec 41-200, Poland; 2 Department of Basic Biomedical Science, School of Pharmacy, Medical University of Silesia in Katowice, ul. Kasztanowa 3, Sosnowiec 41-200, Poland; 3 Department of Biochemistry, School of Medicine with the Division of Dentistry, Medical University of Silesia in Katowice, ul. Jordana 19, 41-808 Zabrze, Poland

**Keywords:** Hyperspectral imaging, Image processing, Measurement automation, Segmentation

## Abstract

**Introduction:**

Hyperspectral imaging has been used in dermatology for many years. The enrichment of hyperspectral imaging with image analysis broadens considerably the possibility of reproducible, quantitative evaluation of, for example, melanin and haemoglobin at any location in the patient's skin. The dedicated image analysis method proposed by the authors enables to automatically perform this type of measurement.

**Material and method:**

As part of the study, an algorithm for the analysis of hyperspectral images of healthy human skin acquired with the use of the Specim camera was proposed. Images were collected from the dorsal side of the hand. The frequency *λ* of the data obtained ranged from 397 to 1030 nm. A total of 4'000 2D images were obtained for 5 hyperspectral images. The method proposed in the paper uses dedicated image analysis based on human anthropometric data, mathematical morphology, median filtration, normalization and others. The algorithm was implemented in Matlab and C programs and is used in practice.

**Results:**

The algorithm of image analysis and processing proposed by the authors enables segmentation of any region of the hand (fingers, wrist) in a reproducible manner. In addition, the method allows to quantify the frequency content in different regions of interest which are determined automatically. Owing to this, it is possible to perform analyses for melanin in the frequency range *λ*_*E*_∈(450,600) nm and for haemoglobin in the range *λ*_*H*_∈(397,500) nm extending into the ultraviolet for the type of camera used. In these ranges, there are 189 images for melanin and 126 images for haemoglobin. For six areas of the left and right sides of the little finger (digitus minimus manus), the mean values of melanin and haemoglobin content were 17% and 15% respectively compared to the pattern.

**Conclusions:**

The obtained results confirmed the usefulness of the proposed new method of image analysis and processing in dermatology of the hand as it enables reproducible, quantitative assessment of any fragment of this body part. Each image in a sequence was analysed in this way in no more than 100 ms using Intel Core i5 CPU M460 @2.5 GHz 4 GB RAM.

## Introduction

The use of imaging in dermatological diagnosis is currently a very rapidly growing branch of medicine and computer science. Computer-assisted medical diagnosis gives much wider possibilities than the methods of traditional evaluation performed by a medical diagnostician. The results obtained from computer analysis are automatic, reproducible, calibrated and independent of human factors related to both the patient and the medical diagnostician who performs the examination. The most common methods of imaging are infrared and visible light. In this respect, the methods of assessment of various types of dermatological conditions based on simple methods of image analysis and processing are extremely popular. In thermal imaging, temperature changes resulting from the imaged diseases such as melanoma as well as thermal effects of the performed dermatological treatments are analysed automatically. As for imaging in visible light, a group of methods which enable morphometric measurements or profiled methods of image analysis and processing are used, for example, to determine the brightness of the RGB components in a segmented skin area. Hyperspectral imaging, which is also used in dermatology, offers much wider capabilities. Multispectral images are acquired using profiled multispectral cameras working in different spectra ranges. Additionally, depending on the frequency and spectrum range, various types of illuminators are used. Matching these two elements (camera and illuminator) is extremely important because of the need to obtain a flat spectrum of the illuminator (lamp) in the range covered by the camera. In this range of light, for example, the visible one, it is possible to observe melanin or haemoglobin at a desired location on the skin. This observation is associated with image analysis enabling automatic and reproducible measurements independent of interindividual variability.

The methods of image analysis and processing used in hyperspectral imaging of any object (not necessarily a medical one) can be divided into several groups, namely morphological methods [1-6], statistical methods [7-9] and profiled algorithms for selected applications. Among the morphological methods, there are classical approaches [1,2,4] and those profiled to the analysis of image sequences [5,6]. The statistical methods are dominated by texture analysis [7-9] which is used as a set of features for classification and recognition. So far, profiled algorithms have been applied to face recognition [10], analysis of skin areas [11], and others [12]. These methods mainly dominate in the segmentation of specific objects [13]. On the basis of segmented objects, their morphometric measurements or analysis of their texture are performed [9]. These are, for example, methods which enable to assess the difference in absorption of radiation by skin chromophores such as haemoglobin or melanin.

The amount and distribution of melanin is in fact an important factor determining, inter alia, the efficacy and safety of treatments in aesthetic medicine with the use of lasers and ILP. In hyperspectral imaging of melanin and haemoglobin, only qualitative analyses of the results obtained are known [14-16]. Therefore, there is a need to objectify the results obtained and to propose fully-automatic measurements of progression and changes of melanin or haemoglobin in the skin of the hand.

The aim of the analysis is to determine melanin and haemoglobin quantity in selected areas of the right hand by using hyperspectral imaging.

## Material

As part of the study, an algorithm for the analysis of hyperspectral images of human, healthy skin acquired with the use of the Specim PFD-V10E camera was proposed. Images were derived from the human hand having 2 Fitzpatrick skin phototypes. Individual hands were illuminated with a typical lamp with flat spectral characteristics in the required range (based on HgAr emission for the VNIR spectral range). The images were obtained retrospectively during routine medical examinations carried out in accordance with the Declaration of Helsinki. As for the described algorithm, no studies and experiments were carried out on humans. The resulting data were anonymized and stored in the output format, source 'dat' (ENVI File). The frequency *λ* of the data obtained ranged from 397 to 1030 nm. Each image was recorded every 0.79 nm, which in total gave 800 2D images for each patient. The resolution *M* × *N* (number of rows and columns) of each image for the selected frequency was 899 × 1312 pixels. For the fixed distance of the object from the camera and the set focusing parameters, there was a square area covering the range 130 × 130 μm per each pixel. A total of 4'000 2D images were obtained for 5 hyperspectral images. These images are subject to further analysis.

## Method

### Preprocessing

**Figure 1:**
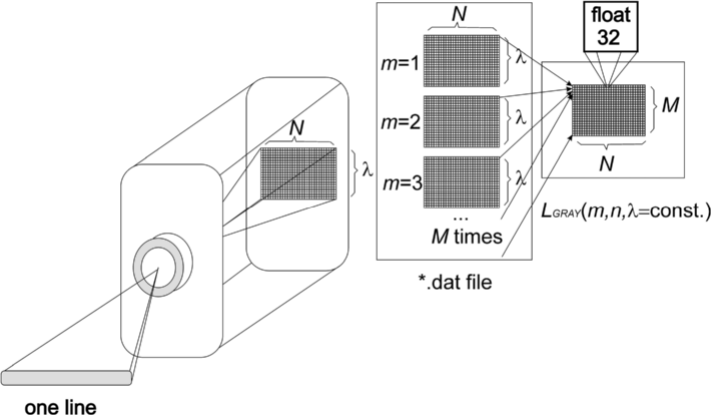
Diagram of the acquisition and organization of data *.dat. The various stages of data analysis and reorganization are performed automatically. A hyperspectral camera saves each row sequentially for individual wavelengths *λ*. In subsequent stages of processing, they are converted to the image *L*_*GRAY*_(*m*,*n*,*λ*). The resulting image is further subjected to further processing steps.

Input files with the extension *.dat containing a sequence of images are reorganized in the preliminary stage. This process (Figure 1) concerns the change in the organization of individual rows and columns obtained for different wavelengths to the sequence of images *L*_*GRAY*_(*m*,*n*,*λ*), where *m* and *n* are row column coordinates and the wavelength *λ* respectively, for which the image was acquired. The number of rows and columns and the wavelength for each image are automatically read from the file *.hdr. This file also contains information about the data format (e.g. data type 4 - float 32 bits) and other elements that need to be taken into account when reading such data (wavelength units, default bands, sensor type, interleave). For the analysed data, the image resolution *M* × *N* was 899 × 1312 pixels.

Each of the images *L*_*GRAY*_(*m*,*n*,*λ*) is calibrated using the reference rows (used for the calibration for a given range of the spectrum *λ*) [17-19], and then subjected to filtration thus forming a new sequence of images *L*_*M*_(*m*,*n*,*λ*). This is median filtration with a mask *h*_*M*_ sized 3 × 3 pixels. The mask size was chosen arbitrarily taking into account the possible contamination of the optical path as well as the image resolution and the size of the object [20-22]. A larger size of the mask *h*_*M*_ caused the removal of not only noise but also small elements in the image which are substantively important. Consequently, the size of 3 × 3 pixels remained. The final step of image pre-processing involves normalization and removal of uneven illumination. In the case of normalization of brightness levels, the range of minimum and maximum values in the image *L*_*M*_(*m*,*n*,*λ*) is extended to the full range of brightness from 0 to 1, that is, the image after normalization *L*_*P*_(*m*,*n*,*λ*) is equal to:

(1) $$L_{P}\left(m,n,\lambda \right)=\frac{L_{M}\left(m,n,\lambda \right)-\min_{m,n}\left(L_{M}\left(m,n,\lambda \right)\right)}{\max_{m,n}\left(L_{M}\left(m,n,\lambda \right)-\min_{m,n}\left(L_{M}\left(m,n,\lambda \right)\right)\right)}$$

It should be noted that this normalization also covers the area to be calibrated. For this reason, the maximum brightness value equal to “1” after normalization corresponds to 100% emission for the wavelength *λ*. Removal of uneven illumination is related to the subtraction from the image *L*_*P*_(*m,n,λ*) the image resulting from its filtration, i.e.:

(2) $$\begin{array}{l} L_{C}\left(m,n,\lambda \right)=L_{P}\left(m,n,\lambda \right)\\ \;-\frac{1}{M_{h2}\cdot N_{h2}}\sum_{m_{2}=1}^{M_{h2}}\sum_{n_{2}=1}^{N_{h2}}\left(L_{P}\left(m+m_{2}-\frac{M_{h2}}{2},n+n_{2}-\frac{N_{h2}}{2},\lambda \right)\cdot h_{2}\left(m_{2},n_{2}\right)\right) \end{array}$$

for *m*∈(*M*_*h2*_/2, *M*_*C*_-*M*_*h2*_/2) and *n*∈(*N*_*h2*_/2, *N*_*C*_-*N*_*h2*_/2).where:

*M*_*h2*_ × *N*_*h2*_ resolution of the mask *h*_2_,

*L*_*C*_(*m*,*n*,*λ*) – output image after the removal of uneven illumination.

To acquire the output image *L*_*C*_(*m*,*n*,*λ*), its convolution with the mask *h*_2_ sized *M*_*h2*_ × *N*_*h2*_ equal to 30 pixels was used. The mask size was selected on the basis of twice the maximum size of the elements, objects visible in the image *L*_*C*_(*m*,*n*,*λ*), in this case it is 15 × 15, i.e. *M*_*h2*_ × *N*_*h2*_ = 30 × 30 pixels. Next, based on the images *L*_*C*_(*m*,*n*,*λ*) and *L*_*P*_(*m*,*n*,*λ*), the appropriate stages of image analysis and processing are carried out.

### Image processing

The images *L*_*C*_(*m*,*n*,*λ*) and *L*_*P*_(*m*,*n*,*λ*) resulting from the initial stage of image processing are used for further processing steps. The image *L*_*C*_(*m*,*n*,*λ*) is further used in order to isolate the region and the object of interest. The image *L*_*P*_(*m*,*n*,*λ*) is a measurable image with respect to the value *λ*.

**Figure 2:**
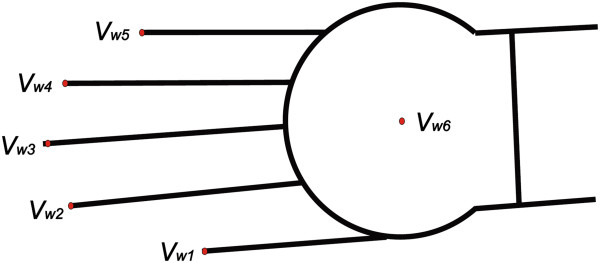
**Hand pattern used during matching to hyperspectral images.** The division into individual (points) areas from *V*_*w*1_ to *V*_*w*6_ is performed automatically. For each area, a number of the finger from *V*_*w*1_ to *V*_*w*5_ and the centre of mass of the wrist *V*_*w*6_ are assigned. In the upper part of the image, there is the brightness pattern sized 100 × 1321 pixels. The distribution and size of the fingers is carried out based on anthropometric data.

Since the aim of the analysis is to determine melanin and haemoglobin quantity in selected areas of the right hand, an important element is the location of characteristic areas. These areas included the individual fingers from *V*_1_ to *V*_5_ (pollex, index, digitus medius, digitus annularis, digitus minimus manus) and the metacarpus and wrist area *V*_6_. Automatic localization of these places (points) from *V*_1_ to *V*_6_ was made based on the pattern shown in Figure 2. Automatic recognition of individual locations (from *V*_1_ to *V*_6_) for each of the patients was carried out based on a hierarchical approach. The image *L*_*C*_(*m,n,λ*) is reduced to a resolution being 10% of the original resolution, that is from the resolution 899 × 1312 pixels to 90 × 131 pixels using the nearest neighbour method. The result is the image *L*_*D*_(*m,n,λ,α*) where *α* is the angle of rotation relative to *L*_*C*_(*m,n,λ*). Then, the angle of the hand inclination *α* is roughly determined, i.e.:

(3) $$\alpha^{*}=\underset{\alpha }{\arg}\left(\max_{n}\left(\sum_{m=1}^{M}\sum_{\lambda }L_{D}\left(m,n,\lambda ,\alpha \right)\right)\right)$$

and *α*∈(0°,180°).

**Figure 3:**
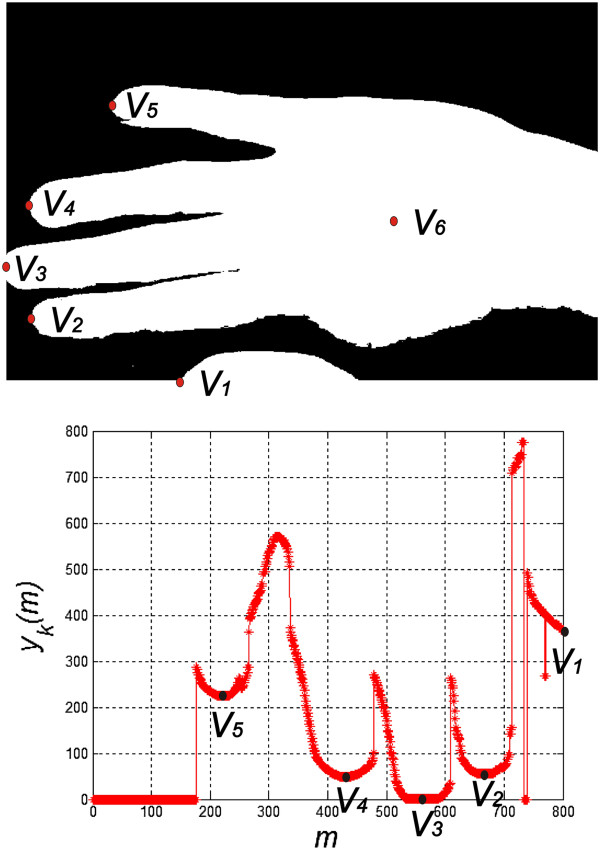
**Binary image of the hand *L***_***B***_**(*m,n*) and the course of the curve *y***_***k***_**(*m*).** The binary image of the hand is acquired in a way which only enables to determine the position of the points *V*_1_ to *V*_6_. These points are determined on the basis of the local minimum in the course *y*_*k*_(*m*). They provide a further basis for matching the pattern.

The obtained result is the value of the rotation angle α* at which the hand image must be rotated for each wavelength *λ* so that it is placed in a horizontal position. The rotation angle is constant for each *λ* so it does not occur in the formula (3). The image *L*_C_^*^(*m,n,λ*) rotated at the angle *α* is subjected to options for finding characteristic points from *V*_1_ to *V*_6_. Detection of the position of characteristic points is carried out based on the local minima found on the contour of the hand - Figure 3. The contour curve *y*_*k*_(*m*) is formed on the basis of the binary image *L*_*B*_(*m,n*) resulting from binarization of the image *L*_*D*_(m,n,λ,α*) for the threshold *p*_*r*_ determined automatically from the Otsu’s formula [23], i.e.:

(4) $$L_{B}\left(m,n\right)=\left\{\begin{array}{ll} 1 & \mathrm{if}\;\sum_{\lambda }L_{D}\left(m,n,\lambda ,\alpha \right)>p_{r}\\ 0 & \mathrm{other} \end{array}\right)$$

The course of the curve *y*_*k*_(*m*) was determined on the basis of the position of the first rows with the pixel values of “1” in the image *L*_*B*_(*m,n*) for each column. Local minima, which are highlighted in Figure 3, were also determined on this basis. In the present case, these are the points *V*_2_ to *V*_6_ (the big finger is not fully visible). The point *V*_6_ is the centre of mass of the wrist calculated on the basis of the image *L*_*O*_(*m,n*) resulting from the opening operation of the image *L*_*B*_(*m,n*), i.e.:

(5) $$L_{O}\left(m,n\right)=\min_{\mathrm{SE}}\left(L_{D}\left(m,n\right)\right)$$

where *SE* – square structural element sized 201 × 201.

The size of the structural element *SE* was selected on the basis of the maximum width of the fingers - for the set distance of the camera and its focal length. In the tests performed, the width of the biggest finger was no more than 200 pixels. On this basis, the centre of mass of the wrist was determined as the coordinates *m*_*v6*_ and *n*_*v6*_ of the point *V*_6_, i.e.:

(6) $$m_{v6}=\frac{\sum_{n}^{N}\sum_{m}^{M}L_{O}\left(m,n\right)\cdot m}{\sum_{n}^{N}\sum_{m}^{M}L_{O}\left(m,n\right)}$$

(7) $$n_{v6}=\frac{\sum_{n}^{N}\sum_{m}^{M}L_{O}\left(m,n\right)\cdot n}{\sum_{n}^{N}\sum_{m}^{M}L_{O}\left(m,n\right)}$$

The centre of mass of the wrist is necessary to roughly detect the position of the hand relative to the pattern. After this stage, the positions of the individual points of fingers, i.e. *V*_1_, *V*_2_, *V*_3_, *V*_4_ and *V*_5_, are matched. Matching is performed on the basis of minimization of the distance between the individual points of the pattern and the analysed image. For the points of the pattern *V*_*w*1_, *V*_*w*2_, *V*_*w*3_, *V*_*w*4_ and *V*_*w*5_, this is minimization of the criterion *J*:

(8) $$J=\sqrt{\sum_{i=1}^{i=5}{\left(m_{\mathrm{vi}}-m_{\mathrm{wvi}}\right)}^{2}+\sum_{i=1}^{i=5}{\left(n_{\mathrm{vi}}-n_{\mathrm{wvi}}\right)}^{2}}$$

where *m*_*vi*_ and *m*_*wvi*_ – coordinates of the points of the analysed image and the pattern *i*∈(1,5).

**Figure 4:**
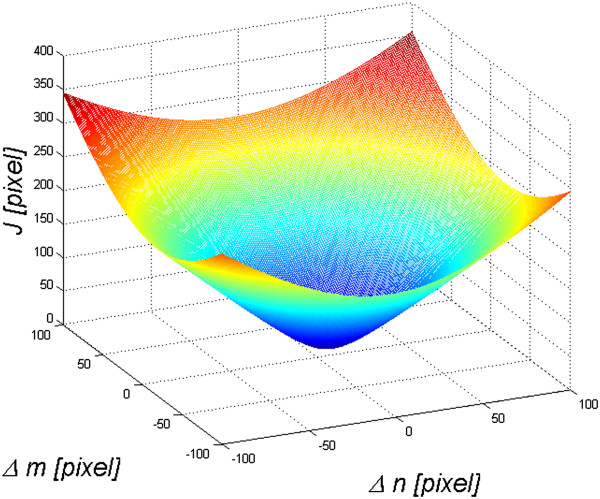
**Graph of changes in the value of the criterion *J *as a function of *Δm *and *Δn*.** Depending on the displacements of the points *V*_1_ to *V*_5_ (*Δm* and *Δn*), the value of the criterion *J* is calculated. One global minimum occurring for the displacements -18 and -3 pixels in the axis of rows and columns respectively is visible. This value provides information about the need to move the image of the patient so that it would better (with respect to the criterion *J*) match the pattern.

**Figure 5:**
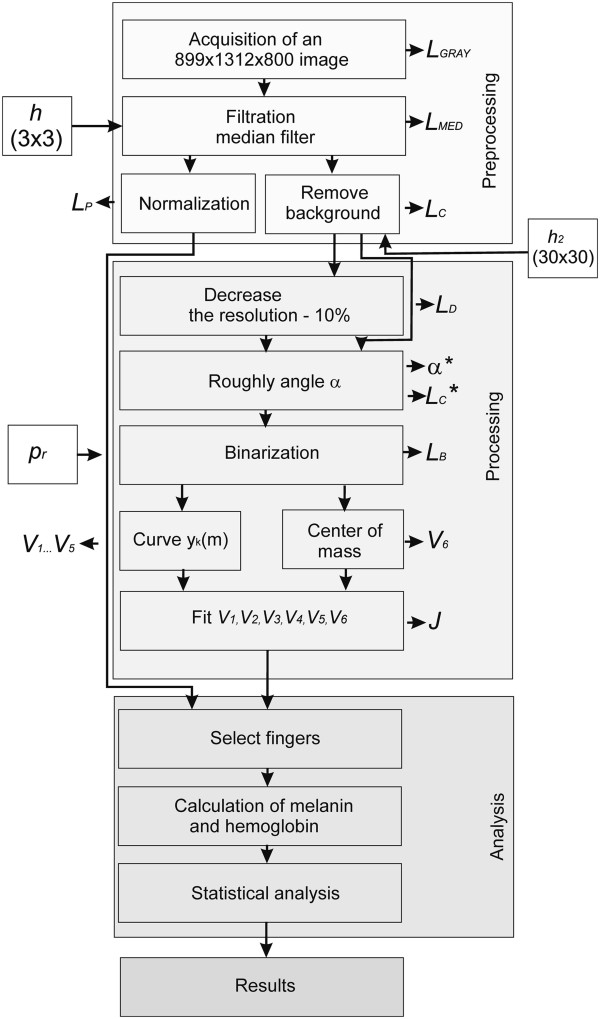
**Block diagram of the proposed algorithm for image analysis and processing.** The subsequent algorithm blocks are profiled to the described issue. In the stage of pre-processing, interference is filtered out of the input image after it is reorganized from the file *.dat to the sequence of images. Next, the image is matched to the pattern for the subsequent frames. In the final stage, it is analysed in specified areas.

**Figure 6:**
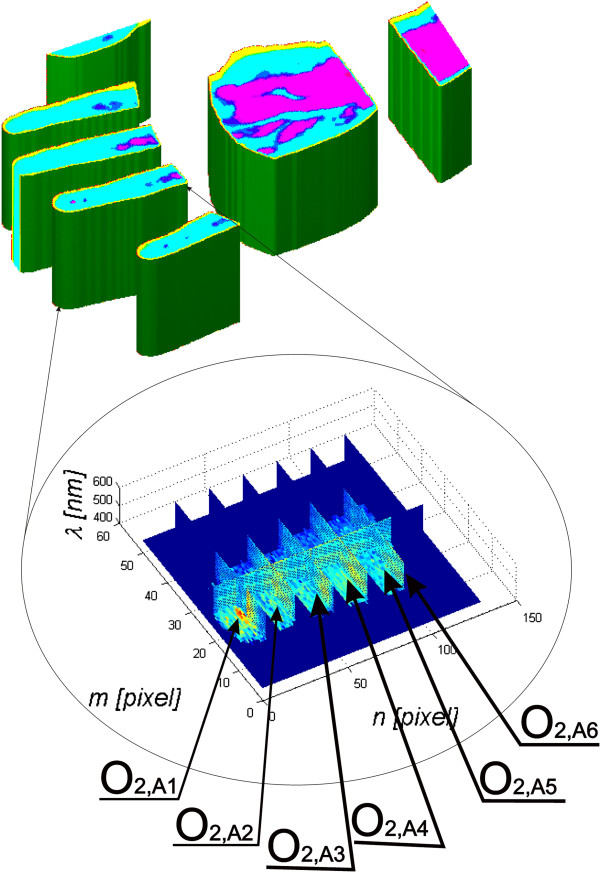
**Contours of melanin and haemoglobin images presented as components R-red and G-green respectively.** For melanin, it is the contour formed as the sum of hyperspectral images in the frequency range *λ*_*E*_∈(450,600) nm, whereas for haemoglobin this is the range *λ*_*H*_∈(350,500) nm. Within these ranges, there are 189 images *L*_P_^*^(*m*,*n*,*λ*) for melanin and 126 images for haemoglobin. The image for melanin and the result of automatic segmentation into characteristic areas, described in the paper, are shown on top and separation into individual objects at the bottom.

The value of the criterion *J* is calculated for the displacement of the points *V*_1_, *V*_2_, *V*_3_, *V*_4_ and *V*_5_ in the range of ±100 pixels in the axis of rows and columns. This range is sufficient for searching for the minimum value of *J*. Examples of the obtained values of the criterion *J* are shown in Figure 4. In this case, displacements amounting to -18 and -3 pixels in the axis of rows and columns respectively were obtained. This value informs about the need to move the image of the patient so that it would better (with respect to the criterion *J*) match the pattern. Final adjustment of the positions of the individual points of the fingers is performed on the basis of an analogous criterion. However, it applies to only one vertex *V*. A block diagram of the proposed algorithm is shown in Figure 5, whereas matching the pattern to the patient's hand is shown in Figure 6. The image *L*_P_^*^(*m*,*n*,*λ*) corrected in terms of affine transformations is further analysed in terms of the content of melanin and haemoglobin.

The performed and described analysis enables automatic and reproducible determination of the position of individual areas of the hand. This process allows to perform further analysis of the brightness of the specific wavelengths *λ*. These lengths correspond to the maximum emissivity of melanin and haemoglobin. For melanin, this is the frequency range *λ*_*E*_∈(450,600) nm and for haemoglobin it is *λ*_*H*_∈(397 nm,500 nm) extending into the ultraviolet. Within these ranges, there are 189 images *L*_P_^*^(*m,n,λ*) for the melanin and 126 images for haemoglobin. Accordingly, the image for melanin *L*_*E*_(*m,n*) was determined as:

(9) $$L_{E}\left(m,n\right)=\sum_{\lambda \in \left(450,600\right)}L_{p}^{*}\left(m,n,\lambda \right)$$

and analogously for the haemoglobin *L*_*H*_(*m*,*n*):

(10) $$L_{H}\left(m,n\right)=\sum_{\lambda \in \left(397,500\right)}L_{p}^{*}\left(m,n,\lambda \right)$$

**Figure 7:**
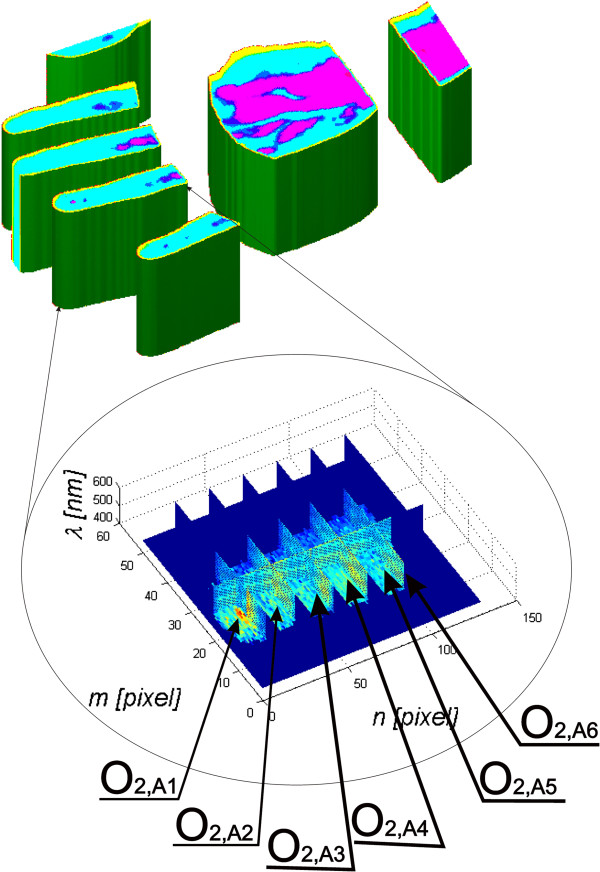
**Result of segmentation for individual wavelengths.** The sample finger (*V*_*2*_) was divided into six areas from *O*_*2,A1*_ to *O*_*2,A6*_ for the left side and from *O*_*2,B1*_ to *O*_*2,B6*_ for the right side. Within these areas, the mean value of melanin and its standard deviation and proportions relative to the other areas are calculated.

The results obtained after brightness normalization are shown in Figure 6. The red component is the image *L*_*E*_(*m*,*n*), while the green component is the image *L*_*H*_(*m*,*n*). The blue component was extinguished. The images *L*_*E*_(*m*,*n*) and *L*_*H*_(*m*,*n*) are the basis for further analysis of the results. Additionally, owing to the applied matching to the pattern, the division into individual fingers and the wrist is further used - Figures 6 and 7.

## Results

**Figure 8:**
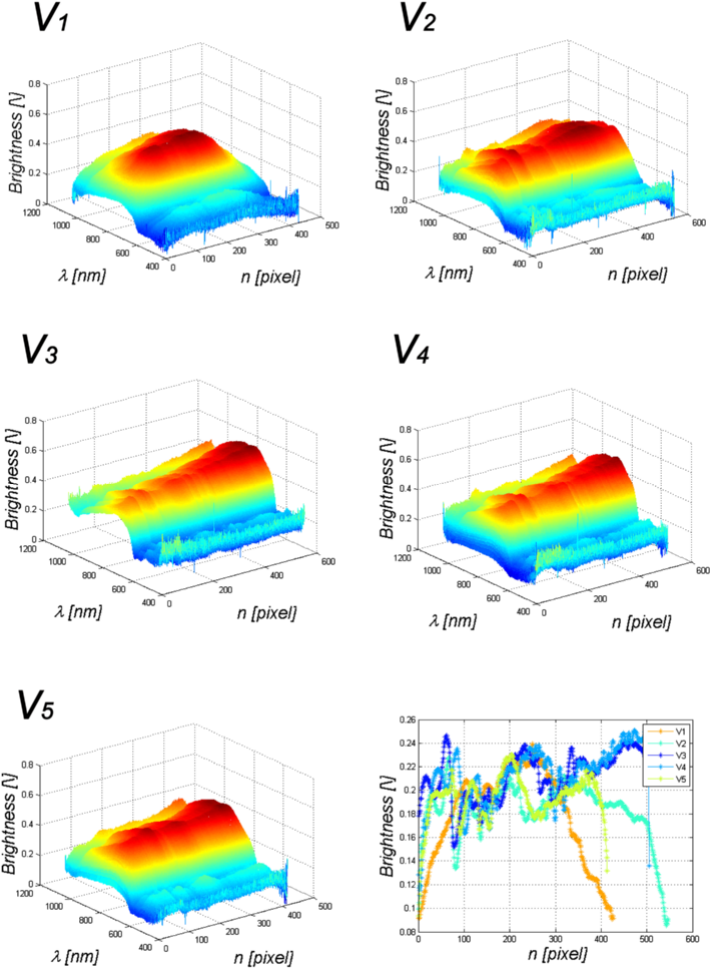
**Graphs of changes in the value of spectrum for wavelengths *λ *for the patient’s subsequent fingers.** The values on the axes are successive columns, wavelength *λ* and brightness for the selected wavelength. The last sixth graph shows the change of melanin along all five fingers of the patient. The *y*-value is the mean value of brightness for *λ*_*E*_∈(450,600) nm. The colours of individual lines correspond to the colours of the fingers shown at the bottom in Figure 6.

The results of automatic division into the fingers and wrist of the human hand (Figure 6) are further used in the tests. These analyses include automatic determination of the changes in the spectrum absorption for different frequencies *λ*∈(397,1030) for individual fingers. To this end, along each finger, the mean along its symmetry axis (for subsequent *n*) for a given frequency *λ* was calculated. The obtained results for the sample calculations of melanin are shown in Figure 8. For subsequent fingers, characteristic changes in the intensity of each frequency *λ* are visible. The last graph in Figure 8 shows changes in melanin along subsequent fingers. When approaching the wrist, an increase in the intensity by about 10% to 20% can be observed. The division of one finger (vertex *V*_*2*_) into different areas is shown at the bottom in Figure 7. The finger was divided into 12 areas, 6 on the side *“A”* and 6 on the side *“B”* of the finger - that is from *O*_*2,A1*_ to *O*_*2,A6*_ for the side *A* and from *O*_*2,B1*_ to *O*_*2,B6*_ for the side *B* respectively - Figure 7. These areas are automatically scaled depending on the size of the finger and other camera settings which may affect the size of the object. The results of the average brightness intensity and standard deviation of the mean for each area are presented in Table 1. Between the sides *A* and *B*, brightness changes are not greater than 1% (*O*_*2,A2,*_*O*_*2,B2*_ and *O*_*2,A3*_, *O*_*2,B3*_). The standard deviation of the mean is the highest for the areas of distal phalanges – areas *O*_*2,A1*_ and *O*_*2,B1*_. The situation is similar for haemoglobin - Table 2. The standard deviation of the mean is a bit smaller than in the case of melanin – it oscillates around the brightness value of 0.02. Higher brightness values exist for other wavelengths not included in the calculation of melanin or haemoglobin - Figure 8.

**Table 1:** **The percentage of melanin for the sample finger (vertex** ***V***_***2***_**) and its standard deviation (STD) of the mean**

	***O***_***2,A1***_	***O***_***2,A2***_	***O***_***2,A3***_	***O***_***2,A4***_	***O***_***2,A5***_	***O***_***2,A6***_	***O***_***2,B1***_	***O***_***2,B2***_	***O***_***2,B3***_	***O***_***2,B4***_	***O***_***2,B5***_	***O***_***2,B6***_
**Brightness**	0.17	0.16	0.16	0.19	0.17	0.16	0.17	0.17	0.17	0.19	0.17	0.16
**STD**	0.06	0.02	0.03	0.02	0.01	0.02	0.05	0.02	0.03	0.02	0.01	0.02

**Table 2:** **The percentage of haemoglobin for the sample finger (vertex** ***V***_***2***_**) and its standard deviation (STD) of the mean**

	***O***_***2,A1***_	***O***_***2,A2***_	***O***_***2,A3***_	***O***_***2,A4***_	***O***_***2,A5***_	***O***_***2,A6***_	***O***_***2,B1***_	***O***_***2,B2***_	***O***_***2,B3***_	***O***_***2,B4***_	***O***_***2,B5***_	***O***_***2,B6***_
**Brightness**	0.17	0.14	0.15	0.16	0.15	0.15	0.17	0.14	0.15	0.16	0.15	0.15
**STD**	0.04	0.02	0.02	0.01	0.01	0.01	0.04	0.02	0.02	0.01	0.01	0.01

### Comparison with other authors’ results

The known results and methods presented by other authors can be divided into:

• Issues related to hyperspectral imaging involving image analysis and processing and

• Methods of analysis and processing of hand images that can be used in hyperspectral imaging.

In the first case, hyperspectral imaging was used, inter alia, to analyse the saturation of HbO_2_ in people of different races (Zuzak [14,15]). Sampling from the hyposthenia region of African-American controls, the area within the square on the image, the percentage of skin HbO_2_ was 77.5 ± 0.2%, which is similar to the skin HbO_2_ percentage of 78.2 ± 0.2% in healthy Caucasian subjects. In contrast, the percentage of skin HbO_2_ in patients was significantly smaller and amounted to 61.0 ± 0.2% (p < 0.001). Percent renal parenchymal oxyhaemoglobin was also analysed by Liu [24]. In [13], there are also other results of analysis which is related only to the analysis of the whole ROI (region of interest) marked manually by an operator. The use of hyperspectral imaging to assess the time of the bruise formation is also interesting – Stam [25]. The inaccuracy found is 2.3% for fresh bruises and 3 to 24% for bruises up to 3 days old. In Authors’ conclusion [25], colour inhomogeneity of bruises can be used to determine their age. For example, the experiment results presented in [13] show that the hyperspectral based method has the potential to identify the spinal nerve more accurately than the traditional method as the new method contains both the spectral and spatial information of nerve sections. There are many other areas of medicine where manual or semi-automatic marking of regions of interest is used - analysis of vibrational Filik J. [26], laparoscopic digital light processing – Olweny EO. [27], blood stains at the crime scene – Edelman G. [28], prostate cancer detection – Akbari H. [29], histopathological examination of excised tissue - Vasefi F. [30] diabetic foot ulcer - Yudovsky D. [31], cancer detection - Akbari H. [32], and others.

In the other case, these are morphological operations used in the classification of various types of artefacts visible in the image [1,2,5,6]. Other methods for classification are also used such as SVM (support vector machines) [4], Gauss-Markov model [7] or wavelet method [9]. In the study of Dicker et al. [16], spectral library was generated with 12 unique spectra, which was used to classify specimens where sample preparation was varied. Using a CT of 0.99 left large areas of the tissues unclassified. Lowering the minimum correlation coefficient to 0.99 enabled all the samples to achieve >85% classification. The results referred mainly to the analysis of a hyperspectral image by analyzing the histograms obtained. Liu Z. et al. [12] propose a novel tongue segmentation method that uses hyperspectral images and the SVM. The presented segmentation of the tongue allows to obtain reproducible and quantitative results. Benediktsson J. et al. [2] present results for a sequential use of morphological opening and closure for the increasing size of the structural element. This methodology is similar to the use of conditional erosion and dilation as in [3]. In turn, Rellier G. et al. [7] propose a probabilistic vector texture model, using a Gauss-Markov random field (MRF). The MRF parameters allow the characterization of different hyperspectral textures.

In conclusion, the well-known studies related to using the methods for the analysis and processing of images into hyperspectral images is dominated by morphological analysis. Segmentation into specific areas refers to simple objects such as the tongue. In each case the methods described are profiled to a particular application. Therefore, the approach to the analysis of the hand proposed in this paper is an extension of these methods into a new area and new dedicated analysis methodology.

## Summary

The paper presents a method for the analysis of melanin and haemoglobin in the area of the human hand. The characteristics of the described method are as follows:

• Repeatability of measurements owing to limiting of operator’s participation in the study,

• Full automatic operation of the algorithm - the arguments for each algorithm function are set once when first starting the algorithm – as they only depend on the type of the multispectral camera used,

• Possibility of any quantitative (not qualitative) assessment of the amount of melanin or haemoglobin in any area of the hand,

• Possibility of automatic comparison of the results of any area of the finger with other areas or other study of the same person in therapy/disease monitoring,

• Time analysis of a single image sequence does not exceed 100 ms when using Intel Core i5 CPU M460 @2.5 GHz 4 GB RAM.

The discussed methodology of hyperspectral image analysis and processing does not fully cover the issue. The presented algorithm for image analysis and processing can also be created by using the techniques from spectral methods [33,34], optical image analysis [35], microscopic analyses [36,37] and others [38-41]. In future work, the authors intend to analyse reproducibility of results for a larger number of patients using different types of hyperspectral cameras operating in the same spectral range. The impact of lighting and calibration method is equally interesting, which will be the subject of the authors’ future work.

## Abbreviations

THV: Thermal camera; STD: Standard deviation; SVM: Support vector machines; ROI: Region of interest.

## Competing interests

The authors declare that they have no competing interests.

## Authors’ contributions

RK suggested the algorithm for image analysis and processing, implemented it and analysed the images. SW, ZW, SK, BBF performed the acquisition of the hyperspectral images and consulted the obtained results. All authors have read and approved the final manuscript.
